# Reflecting on Achievements and Renewing Our Vision

**DOI:** 10.1002/hcs2.127

**Published:** 2025-01-28

**Authors:** Zongjiu Zhang, Tien Yin Wong, Haibo Wang, Jiefu Huang

**Affiliations:** ^1^ Institute for Hospital Management Tsinghua University Beijing China; ^2^ Tsinghua Medicine Tsinghua University Beijing China; ^3^ Research Centre of Big Data and Artificial Intelligence for Medicine The First Affiliated Hospital of Sun Yat‐sen University Guangzhou Guangdong China; ^4^ China National Organ Donation and Transplantation Committee Beijing China

As we bring 2024 to a close and step into the promise of 2025, it is a moment to celebrate our accomplishments, revisit our commitments, and chart the course ahead for *Health Care Science*.

## 1 A Year of Progress

This year marked extraordinary growth for *Health Care Science*. We received a total of 225 manuscript submissions—a remarkable 55.8% increase from the previous year. Thanks to the dedication and expertize of our reviewers and editorial team, we maintained an average review time of just 35 days, ensuring a swift and rigorous editorial process.

Another milestone was our journal's inclusion in prominent indexing platforms such as DOAJ, Scopus, PubMed Central, and ESCI. These achievements enhance the visibility and accessibility of our published work, and we are now preparing to apply for indexing in MEDLINE, further expanding our reach within the global academic community.

## 2 A Broad and Impactful Scope

The breadth of topics covered by *Health Care Science* in 2024 reflects its commitment to interdisciplinary research and its role as a platform for addressing pressing challenges in healthcare.

Post‐pandemic, we took the opportunity to reflect on preparedness and response, publishing impactful papers such as *“Innovative public strategies in response to COVID‐19: A review of practices from China”* [1]. Global health and policy issues remain a central focus, as highlighted by articles such as *“The African Medicines Agency and Medicines Regulation: Progress, challenges, and recommendations”* [2], which explores the evolving role of regulatory frameworks in medicines access. Our commitment to improving healthcare management and practices is evident in studies like *“Improving transitional care after acute myocardial infarction: A scoping review”* [3]. Ethical issues were highlighted in discussions of clinical xenotransplantation [4] and systematic reviews of ethical approvals in medical reporting [5], as well as the dedicated editorial to *the 2024 revision of the Declaration of Helsinki (DoH)* [6], indicating a strong focus on the social dimensions of health care.

More importantly, this year, *Health Care Science* put a strong emphasis on innovations in digital health and AI, with articles on the deployment of machine learning in healthcare (*“Toward real‐world deployment of machine learning for health care: External validation, continual monitoring, and randomized clinical trials”* [7]), advancements in medical imaging (*“Leveraging anatomical constraints with uncertainty for pneumothorax segmentation”* [8]), evaluation of large language models in healthcare (*“A systematic evaluation of the performance of GPT‐4 and PaLM2 to diagnose comorbidities in MIMIC‐IV patients”* [9]), and the broader impact of modern technologies on healthcare systems (*“Revolutionizing healthcare and medicine: The impact of modern technologies for a healthier future—A comprehensive review”* [10]).

These contributions—spanning health policy, technology, and practice—have been among our most read and most cited articles, underscoring our commitment to addressing contemporary challenges while advancing healthcare knowledge. Together, they highlight *Health Care Science*'s pivotal role in fostering innovative and impactful research on a global scale.

## 3 Revisiting Our Promises

When we launched *Health Care Science*, we set out with bold ambitions to redefine what a health care journal could be—to address the pressing gaps in health care systems research that other journals often overlook. However, as with any endeavor, we recognize that growth comes with continuous reflection and adjustment. One key lesson we've learned this year is that certain practices—such as free‐format submission—can inadvertently burden our reviewers, despite the good intentions behind them. We have taken this feedback into account and revised our submission process to make it more manageable while maintaining high academic standards.

As we move forward, we are also introducing new article types to better serve our mission:

1. Brief Reports (replacing case reports)—These focus on non‐clinical reports of inspiring healthcare management cases that offer valuable insights and practical lessons for the broader health care community.
2. News and Views—A fast‐track publishing category to highlight the most up‐to‐date academic advances, including new research findings and selected works from top‐tier conference proceedings.
3. Practice and Policy—This category allows for free‐format submissions tailored to the case presented, showcasing evidence‐based, state‐of‐the‐art knowledge on specific aspects of practice and policy, or newly developed management modes that warrant attention.

These changes reflect our commitment to evolve with the needs of the healthcare field, ensuring *Health Care Science* remains a timely and relevant platform for innovative research and thought leadership.

## 4 Looking Ahead

As we prepare for the challenges and opportunities of 2025, we renew our commitment to excellence, innovation, and inclusivity. Our ambition remains to create a journal that not only informs but also inspires meaningful change in health care systems worldwide. We invite our community to continue this journey with us—authors, reviewers, and readers alike. Together, we can further elevate the impact of *Health Care Science* and contribute to shaping a brighter future for health care.

Thank you for being an integral part of our journey. May 2025 bring us new opportunities, achievements, and moments of joy.


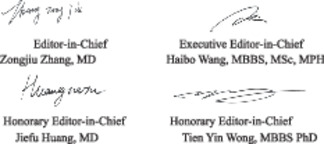


## Author Contributions

**Zongjiu Zhang:** conceptualization (lead), writing–original draft (lead). **Tien Yin Wong:** conceptualization (equal), writing–review and editing (equal). **Haibo Wang:** writing–review and editing (equal). **Jiefu Huang:** supervision (equal).

## Ethics Statement

The authors have nothing to report.

## Informed Consent

The authors have nothing to report.

## Conflicts of Interest

The authors declare no conflicts of interest.

## Data Availability

Data sharing is not applicable to this article as no data sets were generated or analyzed during the current study.
